# Stability of Hairline Lowering Using Small Footprint Soft Tissue Anchoring Devices

**DOI:** 10.1002/lary.70207

**Published:** 2025-10-21

**Authors:** Cyrus W. Abrahamson, Hasan Pracha, Jaymie A. Bromfield, Douglas M. Sidle, James C. Wang

**Affiliations:** ^1^ Northwestern University Feinberg School of Medicine Chicago Illinois USA; ^2^ Division of Facial Plastic and Reconstructive Surgery, Department of Otolaryngology—Head and Neck Surgery Northwestern University Chicago Illinois USA

**Keywords:** aesthetic procedure, hairline lowering, outcomes, soft tissue anchor

## Abstract

**Objectives:**

One principle of facial aesthetics is based on “facial thirds,” and hairline recession/large foreheads disrupt this balance. Hairline lowering can improve harmonious aesthetics and often uses bioabsorbable implants. This study seeks to establish the outcomes and stability of hairline lowering using the Endotine Forehead‐mini in a single‐surgeon cohort in the United States.

**Methods:**

This is a retrospective review of a single‐surgeon cohort from 2011 to 2024 that underwent hairline lowering. Patient demographics, outcomes, and complications were documented. Upper facial third ratios were measured preoperatively and postoperatively and compared using paired *t* tests. The stability of the upper facial third ratio was assessed over time using paired *t* tests between the first postoperative visit and subsequent visits.

**Results:**

100 patients were included, with 58 having images available for analysis. 100% of patients had a statistically significant reduction in upper third percentage medially (*μ* = 3.5% lower, 95% CI = 3.1%–3.8%, *p* < 0.001) and laterally (*μ* = 3.1% lower, 95% CI = 2.7%–3.6%, *p* < 0.001) at their first postoperative visit. As patients travel for this procedure, follow‐up varied; however, hairline lowering was stable from first follow‐up to 3–6 months, 6–12 months, and past 12 months. Patient satisfaction appeared high but was not formally assessed. Complications occurred in 26% of patients: transient alopecia in 14% (all resolved with minoxidil), 10% required additional surgery (scar revision, further hairline lowering, or hematoma evacuation), 2% had hematomas managed nonsurgically, 5% had scalp irritation, and 1% had a postoperative infection (treated with antibiotics).

**Conclusion:**

Use of the Endotine Forehead‐mini was associated with substantial, sustained hairline lowering with minimal risks.

**Level of Evidence:**

4.

## Graphical abstract

Facial aesthetics and balanced proportions are influenced by “facial thirds,” and hairline recession/large foreheads can alter this natural harmony. Hairline lowering with bioabsorbable fixation devices, such as the Endotine Forehead‐mini, aims to restore facial balance through stable advancement of the hairline. In this 2011–2024 single‐surgeon series of 100 patients, hairline lowering with the use of these implants produced significant and lasting reductions in upper facial height with low complication rates and high patient satisfaction.

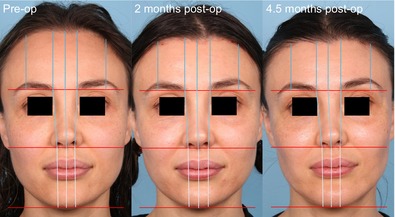

## 1 Introduction

The face is crucial in determining the physical appearance of individuals [1]. Facial thirds, dividing the face into three equal horizontal areas—from trichion to glabella, glabella to subnasale, and subnasale to menton—comprise a significant part of facial aesthetics [2]. A preeminent determinant of facial thirds is hairline height, linked with forehead size. In recent years, the importance of an appropriately proportional hairline has been discussed at length, with high hairlines resulting from a variety of causes including hormonal changes, stress, traction, trauma, facial surgeries such as brow lifts, medication, or diet changes [3].

Procedures aimed at correcting high hairlines include brow lifts, hair transplantation, and hairline lowering. In more recent years, hairline lowering surgery has become increasingly popular as it offers a strong alternative to correcting disproportionately large foreheads with low complication rates by directly excising forehead skin [2, 4, 5]. This approach provides unmatched hair density and immediate results, equivalent to the transplantation of 7000–9000 grafts [6]. Ideal candidates should have a stable frontal hairline, excluding many men, and a moderate to highly mobile scalp, allowing for adequate advancement to justify this more invasive method [7].

While hairline lowering surgery can be conducted by simply suturing after excising the excess skin, facial plastic surgeons often utilize soft tissue anchors to help maintain the desired lowering. In 2009, Ramirez et al. introduced the single‐staged Endotine‐assisted hairline lowering, and it has remained a cornerstone technique and mainstay of care [8]. The use of Endotine devices (MicroAire Surgical Instruments, Charlottesville, VA), absorbable clips that fixate the forehead after scalp skin excision, eliminates the need for screw removal. Since 2009, Endotine devices have become broadly used, and additional investigations, such as those by Lee et al. and Kim et al. in Korea, have shown that hairline lowering using Endotine devices provides substantial lowering that is stable over time [5, 9].

However, there are several areas requiring further investigation. First, no new studies have investigated outcomes of hairline lowering using Endotine devices in the United States. Second, while the original Endotine devices are still commonly used, a new Endotine technology has emerged: the Endotine Forehead‐mini, a lower profile polydioxanone suture (PDS) anchoring fixation from the same system. Finally, while hairline lowering has been shown to provide substantial lowering, no studies have investigated hairline lowering in terms of facial thirds. While every study on hairline lowering mentions the importance of facial thirds, they have not reported how facial thirds change following surgery. Therefore, this study seeks to address these gaps by investigating outcomes and stability of hairline lowering surgery using the Endotine Forehead‐mini in a US cohort.

## 2 Materials and Methods

### 2.1 Patient Cohort

Following Institutional Review Board approval (IRB STU00211795), this study retrospectively reviewed charts from a single‐surgeon cohort to identify patients who had undergone hairline lowering from 2011 to 2024 utilizing the Endotine Forehead‐mini soft tissue anchoring device. Patients were excluded if they did not undergo hairline lowering with the Endotine Forehead‐mini soft tissue anchor or were a minor (< 18). While patients undergoing brow lifts were not excluded, none in this cohort had one, allowing for isolation of the effects of hairline lowering with the Endotine Forehead‐mini.

All patients who underwent hairline lowering did so under a technique based on that described by Kabaker and Champagne [10]. Specifically, a peritrichial trichophytic incision one to two mature hairs into the hairline with an undulating pattern was made, followed by subgaleal dissection and undermining to 2 cm above the brow. Posterior subgaleal dissection proceeded over the vertex to the occiput. Then, the degree of hairline advancement and the amount of brow skin that could be excised was determined with a D'Assumpcao forceps. Excess skin was excised, and the scalp was advanced. Two Endotine Forehead‐minis were placed into frontal bone at the mid‐pupillary line bilaterally just behind the leading edge of the hair‐bearing flap and tacked to the galea prior to closure. This reduces tension on the wound closure and ensures more hairline advancement and less brow lift.

### 2.2 Data Collection and Analysis

Utilizing the identified patient population, chart review was conducted with collection of patient demographics, facial and hairline characteristics, surgical information, complications and side effects, and patient follow‐up information. Additionally, a subset of patients had preoperative and postoperative images taken utilizing standard FPRS imaging techniques. For patients to be included for image analysis, they were required to have complete frontal photos with both their medial and lateral forehead visible for facial third ratio measurements. For these patients, images from several time frames were collected, including preoperatively and postoperatively. If patients had more than one postoperative visit, their postoperative visits were divided into time frames, including within 3 months of surgery, 3–6 months, 6–12 months, and 12+ month time frames.

**FIGURE 1:**
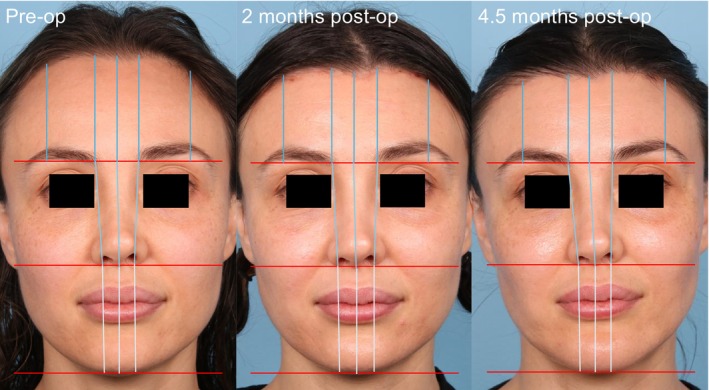
Representative patient demonstrating facial thirds measurements. This patient had a 3.2% lowering medially and 3.1% lowering laterally at 2 months and 3.9% lowering medially and 3.5% lowering laterally at 4.5 months, exhibiting hair regrowth and scar maturation.

Instead of utilizing absolute measurements of hairline lowering, this study elected to utilize facial thirds ratios. This decision was made as patients have different desires and needs for hairline lowering, and by utilizing ratios instead of absolute values, the amount of lowering and final outcomes are more standardizable between patients. Facial thirds from each image were measured using the Fiji software, and facial thirds were measured medially and laterally to better differentiate the impact of hairline lowering on different parts of the hairline [11]. As shown in Figure 1, patients had their facial thirds measured in three places medially: from the trichion to glabella, glabella to subnasale, and subnasale to menton. These measurements were averaged for each third, and then each third was added together for a total face length. Using this total length, the lower, middle, and upper facial third ratios could be calculated. For the medial upper facial third ratio, the trichion‐to‐glabella measurement was used. While this was used for the medial upper facial third ratio, the lateral upper facial third was measured from the trichion to the level of the glabella along a vertical line passing through the lateral canthus. Measurements were taken bilaterally and averaged to account for any right–left differences. For analysis of the medial‐to‐lateral forehead ratio, comparisons were made between preoperative and postoperative images to determine whether hairline lowering was uniform. In patients with more than one postoperative visit, the ratio was additionally compared between the first and last available postoperative images to assess stability over time.

### 2.3 Statistical Analysis

Statistical analysis was performed utilizing RStudio version 4.4.0. Paired *t* tests were performed between preoperative and postoperative upper facial thirds for both medial and lateral measurements, and patients with more than one postoperative visit had paired *t* tests conducted between their first postoperative visit and later time points of interest. Shapiro–Wilks tests were used to confirm that paired *t* tests were applicable in all cases. Two‐sided *p* values were used for all analyses, with the alpha criterion set at 0.05.

## 3 Results

### 3.1 Patient Population

Patient demographics are outlined in Table 1. A total of 117 patients were identified as having undergone hairline lowering surgery from 2011 to 2024, with 100 patients having undergone surgery with the Endotine Forehead‐mini. These 100 patients had a mean age of 31.7 ± 9.6 years, and 95 patients (95%) were women. A total of 73 patients (73%) were White. No patients were transgender and undergoing hairline lowering for gender‐affirming purposes. The most common cause of a high hairline was idiopathic or congenital, accounting for 89 patients (89%). This included individuals born with a high hairline but normal hair density or with no identifiable cause apart from alopecia. The next most common cause was traction alopecia, affecting 6 patients (6%).

**TABLE 1:** Demographic and clinical information of entire patient cohort.

Characteristic	*N* = 100
Age	31.7 ± 9.6
*Sex*	
Female	95
Male	5
*Race*	
White	73
Black	13
Hispanic	8
Asian or Pacific Islander	5
Other	1
*Cause of hair loss*	
Idiopathic/congenitally high hairline	89
Traction alopecia	6
Aging	2
Prior surgery	2
Other known cause	1

### 3.2 Outcomes and Complications

While this study did not use any validated patient‐reported outcome measure, patient satisfaction appeared to be high based on available chart documentation. A total of 26 patients (26%) experienced a postoperative complication or side effect. Transient alopecia was the most common, occurring in 8 patients (8%) as an isolated finding and in an additional 6 patients (6%) who also had other complications (4 patients [4%] who underwent scar revision, 1 patient [1%] who underwent further hairline lowering, and 1 patient [1%] with scalp irritation). All cases of transient alopecia were successfully treated with topical minoxidil. Additional surgery was required for 10 patients (10%), including 6 patients (6%) who underwent scar revision, 3 patients (3%) who underwent further hairline lowering, and 1 patient (1%) who required evacuation of a postoperative hematoma. An additional 2 patients (2%) developed hematomas that were managed nonsurgically. Scalp irritation occurred in 5 patients (5%). 1 patient (1%) developed a postoperative infection that resolved with antibiotic treatment.

### 3.3 Facial Thirds Ratios

**FIGURE 2:**
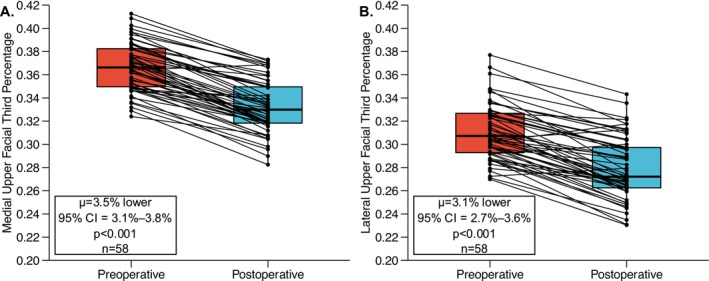
Paired *t* test comparing preoperative and postoperative upper facial third percentages. (A) Medial upper facial third percentages. (B) Lateral upper facial third percentages.

**FIGURE 3:**
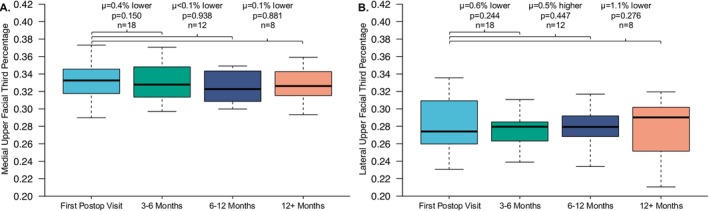
Paired *t* test comparing first postoperative upper facial third percentages with subsequent postoperative upper facial third percentages. (A) Medial upper facial third percentages. (B) Lateral upper facial third percentages.

Of the 100 total patients, 58 had images that could be utilized for photographic analysis. The demographic characteristics of these patients are reported in Table 2. All patients who had preoperative and postoperative images saw a statistically significant reduction in upper facial third percentage medially (*μ* = 3.5% lower, 95% CI = 3.1%–3.8%, *p* < 0.001) and laterally (*μ* = 3.1% lower, 95% CI = 2.7%–3.6%, *p* < 0.001) as visualized in Figure 2. Among these patients, 18 (18%) had more than one postoperative visit. All 18 had follow‐up within 3 months and were subsequently seen at 3–6 months; of these, 12 patients (12%) were followed at 6–12 months and 8 patients (8%) had follow‐up beyond 12+ months. The stability of hairline lowering in these patients who had longitudinal follow‐up is shown in Figure 3, with hairlines remaining stable both medially and laterally. The ratio of medial‐to‐lateral forehead size was unchanged between preoperative and postoperative measurements, indicating uniform hairline lowering (*p* = 0.812). No difference in ratio was found between first and last postoperative visits confirming stability of medial‐to‐lateral forehead ratio over time (*p* = 0.953).

**TABLE 2:** Demographic and clinical information of patients with images.

Characteristic	*N* = 58
Age	30.8 ± 9.8
*Sex*	
Female	55
Male	3
*Race*	
White	42
Black	8
Hispanic	6
Asian or Pacific Islander	2
*Cause of hair loss*	
Idiopathic/congenitally high hairline	51
Traction alopecia	6
Aging	1

## 4 Discussion

This study assessed outcomes and complications of hairline lowering using the newer Endotine Forehead‐mini system based on the Kabaker and Champagne technique in a single‐surgeon cohort in the United States [10]. Consistent with prior work, our study was comprised of mostly women, demonstrating the better candidacy of women for hairline lowering surgeries, whereas men more often present with male pattern hair loss requiring different surgical approaches [7, 12, 13, 14]. The study population was predominantly White, reflecting the demographics of the study center and differentiating from previous investigations which have been predominantly or entirely Asian patients [2, 5, 9, 15]. While these prior studies have provided valuable information, longer‐term outcomes in different patient populations have been lacking. Considering that skin has been shown to have varying biophysical properties depending on ethnicity and geographic inhabitancy, this type of investigation in more diverse patients is essential [16, 17]. Additionally, the perceived ideal facial thirds ratios differ based on culture, with Asians perceiving a smaller upper and lower third as more visually attractive [5, 18]. This can limit conclusions regarding lowering's efficacy in patients who may not have the same aesthetic preferences. Therefore, this study is one of the first steps towards providing US‐based facial plastic surgeons with an understanding of outcomes and stability of hairline lowering with the Endotine Forehead‐mini.

Regarding efficacy, our study demonstrated a significant reduction in the upper facial third ratio, both medially and laterally, showing that hairline lowering surgery is effective in achieving meaningful results. Notably, medial hairline lowering consistently approached the target proportion of approximately 33%, aligning with the desired proportion of facial thirds. Prior studies have demonstrated hairline lowering using absolute measurements in millimeters or centimeters, which, while useful, impedes interpatient comparability [2, 4, 5, 8]. As each patient has unique goals and circumstances, using ratios provides greater insight into achieving aesthetically pleasing results. Overall, these results demonstrate that hairline lowering not only reduces the upper facial third ratio but also brings facial thirds into closer proportion, an adjustment associated with greater facial balance and widely recognized aesthetic ideals.

Consistent with work investigating stability utilizing the original Endotine, medial and lateral hairline reductions remained stable throughout the follow‐up period [5]. This stability highlights the efficacy of the Endotine Forehead‐mini in contributing to long‐term outcomes without significant regression. Compared with the original Endotine, the Endotine Forehead‐mini has a smaller footprint (60% smaller and one‐third the mass), fewer tines (3 vs. 5), and a low‐profile design intended to reduce palpability and visibility, particularly in patients with thinner scalps [19, 20]. To our knowledge, this is the first description of the Endotine Forehead‐mini in the literature, and the results demonstrate that despite its smaller footprint and fewer tines, it effectively facilitates lowering at the site of the excision. Its reduced footprint allows for smaller incisions, quicker absorption, and faster recovery, and, while not formally measured, our experience suggests complete dissolution by 6 months, a markedly faster resorption compared with the 12–18 month absorption window of the original Endotine [21, 22]. Additionally, while palpability, irritation, and discomfort have been reported for the original Endotine, none of our patients reported these issues, likely due to the Endotine Forehead‐mini's small footprint and rapid absorption.

Interestingly, while initial postoperative measurements showed a slightly higher than expected hairline position, further follow‐up revealed that the hairline lowered over time in certain patients, although not significantly. This is likely due to scar maturation and regrowth of hair around the suture line and implants, enhancing the final aesthetic result. Additionally, the medial‐to‐lateral ratio of hairline lowering remained unchanged postoperatively, which is notable for maintaining hairline symmetry and uniformity. This is a positive outcome for patients with uniformly high hairlines, as it ensures proportional reduction across the forehead. Preserving this symmetry contributes to a natural aesthetic and may serve as a key benchmark in evaluating surgical success for hairline advancement.

While the procedure yielded beneficial results, the rate of complications observed was higher than previously reported, with Vila et al. citing complications pooled from meta‐analyses at < 1% [4]. Furthermore, an effectiveness and safety study in Korea utilizing the alternate bone tunneling suture for hairline surgery displayed a lower complication rate, with similar effectiveness [2]. Although no long‐term adverse outcomes were recorded, this association warrants further investigation to determine whether specific side effects are attributable to the Endotine Forehead‐mini or the surgical technique itself. Most complications were minor and included scar revisions or the need for secondary hair transplants. Notably, only one patient required reoperation for a hematoma, while five patients underwent scar revision and four opted for subsequent hair transplants to enhance the aesthetic result. All complications were promptly managed, and no cases resulted in permanent adverse outcomes, underscoring the overall safety of the procedure.

Despite the promising results, this study has limitations that must be acknowledged. It was retrospective in design which limits conclusions that can be drawn. Future prospective investigations are necessary to better understand hairline lowering's effectiveness with and without the Endotine Forehead‐mini. The patient population was relatively homogenous, comprised of predominantly White women. This sets us apart from many prior studies, such as Lee et al., that investigated the original Endotine's effectiveness, but future studies into more diverse populations are required [5, 9]. Additionally, hairline lowering was assessed using facial thirds percentages rather than objective measurements. While this method accounts for individualized aesthetic goals, facilitating interpatient comparison and opening a new avenue for investigation, it does not give an absolute measurement for comparison with prior work. Finally, medial measurements may be more reliable, given the standardized front‐on imaging used for analysis. The curvature of temporal recessions can make lateral measurements less precise, a factor that should be addressed in future studies with advanced imaging techniques.

Several areas require further investigation. While this study recorded patients' demographics, additional work is necessary to assess outcomes by sex and race given the small number of male patients and the diverse etiologies of high hairlines and hair care practices. One important population is Black and African American patients, in whom traction alopecia—common due to hair care practices—can make hairline lowering particularly impactful [23, 24]. While this study had a small number of Black patients, further investigation is essential. Hairline lowering's efficacy for transgender patients also needs investigation as it is frequently used for gender‐affirming care. Additionally, while isolated hairline lowering benefits patients with relatively symmetrical hairlines, its role in managing more complex patterns, such as deep temporal recessions, is less clear [25]. Combining it with hair transplantation may improve outcomes in these cases, and while not well studied, limited work in facial feminization suggests it can address the entire upper third in one operation [26]. Finally, the Endotine Forehead‐mini appeared safe for hairline lowering, with no long‐term complications observed. However, our short‐term complication rate was higher than other studies. Most complications were scar‐related, highlighting the need to identify contributing factors, such as patient postoperative care, to ensure optimal outcomes.

## 5 Conclusion

This study demonstrates that hairline lowering surgery is an effective, stable, and safe procedure for addressing high hairlines, particularly in female patients. This is the first study to show stability of hairline lowering using the Endotine Forehead‐mini, highlighting the benefits of this device in maintaining outcomes. Additionally, this is the first study to utilize facial thirds measurements for hairline lowering, allowing for improved interpatient comparability for future investigations. Hairline lowering should be strongly considered in patients who have stably high hairlines and desire surgical enhancement.

## Conflicts of Interest

The authors declare no conflicts of interest.

## Data Availability

The data that support the findings of this study are available from the corresponding author upon reasonable request.
